# Additively custom‐made 3D‐printed subperiosteal implants for the rehabilitation of the severely atrophic maxilla (a case report)

**DOI:** 10.1002/ccr3.8135

**Published:** 2023-11-06

**Authors:** Mahnaz Arshad, Nourin Khoramshahi, Gholamreza Shirani

**Affiliations:** ^1^ Department of Prosthodontics, School of Dentistry, International Campus Tehran University of Medical Sciences Tehran Iran; ^2^ School of Dentistry, International Campus Tehran University of Medical Sciences Tehran Iran; ^3^ Department of Oral and Maxillofacial Surgery, School of Dentistry Tehran University of Medical Sciences Tehran Iran

**Keywords:** aggressive periodontitis, alveolar bone loss, bone resorption, endosseous dental implantation, subperiosteal dental implantation

## Abstract

**Key Clinical Message:**

Subperiosteal implants might be the future first‐line treatment in patients with compromised alveolar ridges, although the use of proper techniques and pre‐surgical imaging is required to ensure treatment success.

**Abstract:**

Severe bone loss puts the success of endosseous implants at risk. This technical report aims to introduce the subperiosteal implants (SPIs) created through additive manufacturing. A case study is presented, outlining the process and strategies employed to fully restore a maxillary structure using a customized subperiosteal implant. The patient, who had previously faced disappointment with traditional endosseous implants, received a customized SPI. A detailed 3‐year follow‐up is also provided. The design of the subperiosteal framework and abutments is based on digital records of the patient's jaw structure and a radiographic stent during occlusion. This ensures optimal placement within the dental arch. The implant and abutments are then three‐dimensional (3D) printed using a titanium alloy, while a provisional denture is 3D‐printed using polymer materials. SPIs offer a viable alternative for individuals with severe jaw bone degeneration, as demonstrated in this report detailing their application in complete maxillary restoration. This patient‐specific, prosthesis‐driven approach avoids the need for bone grafting and enables immediate functional recovery through a single surgical procedure.

## Graphical abstract

Printed maxillary subperiosteal implanted.

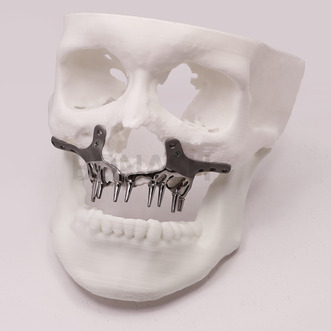

## 1 INTRODUCTION

Dental endosseous implants present a favorable option for tooth replacement in numerous cases of edentulism. They boast a remarkable success rate and yield consistent outcomes. Several studies suggest that the success rate of endosseous dental implants could potentially range from 89% to 99%.^1^ However, endosseous implants require a certain amount of bone quantity and quality to succeed, and their ideal requirements are not always available. In complex situations involving resorbed or compromised alveolar bone, the process of endosseous implantation becomes intricate and challenging.^2^ Conversely, a significant concern regarding endosseous dental implants is peri‐implantitis, which impacts around 20% of patients within the initial 5–10 years following surgery, as indicated by studies. This matter not only jeopardizes the implant's long‐term viability but also diminishes the overall bone quality, making implant replacement a challenging endeavor.^3^ Several strategies have been suggested aiming to induce bone regeneration in cases lacking sufficient bone tissue. However, each method comes with many additional risks and complications. Bone grafts are the most adopted strategy to replace the resorbed alveolar bone. However, this treatment has many downsides, such as its complexity, long healing period, increased risk of complications, patient discomfort in extraoral grafts, and limited bone supply in intraoral grafts.^4^, ^5^

Subperiosteal implants (SPIs) were first introduced in the 1940s but were soon eliminated due to prevalent complications. The first models of subperiosteal implants were custom‐made cobalt‐chrome or titanium implants placed below the periosteum which held the prosthesis in place. These implants lacked adequate fitting and often caused peri‐implantitis by implant movements.^1^, ^6^

In the past decade, the concept of subperiosteal implants has reemerged with the evolving digital technologies used in dental and prosthesis fabrications. Techniques involving computed tomography (CT) and CBCT scans have played a significant role in this resurgence. The utilization of additive manufacturing technology in the fabrication of SPIs has notably improved the precision of fit, resulting in a substantial increase in bone‐to‐implant contact. This advancement has consequently led to a significant reduction in the occurrence of implant failures.^2^, ^6^

This article highlights a case involving the replacement of failed endosseous implant‐supported prostheses with custom‐made subperiosteal implants.

## 2 CASE REPORT

**FIGURE 1:**
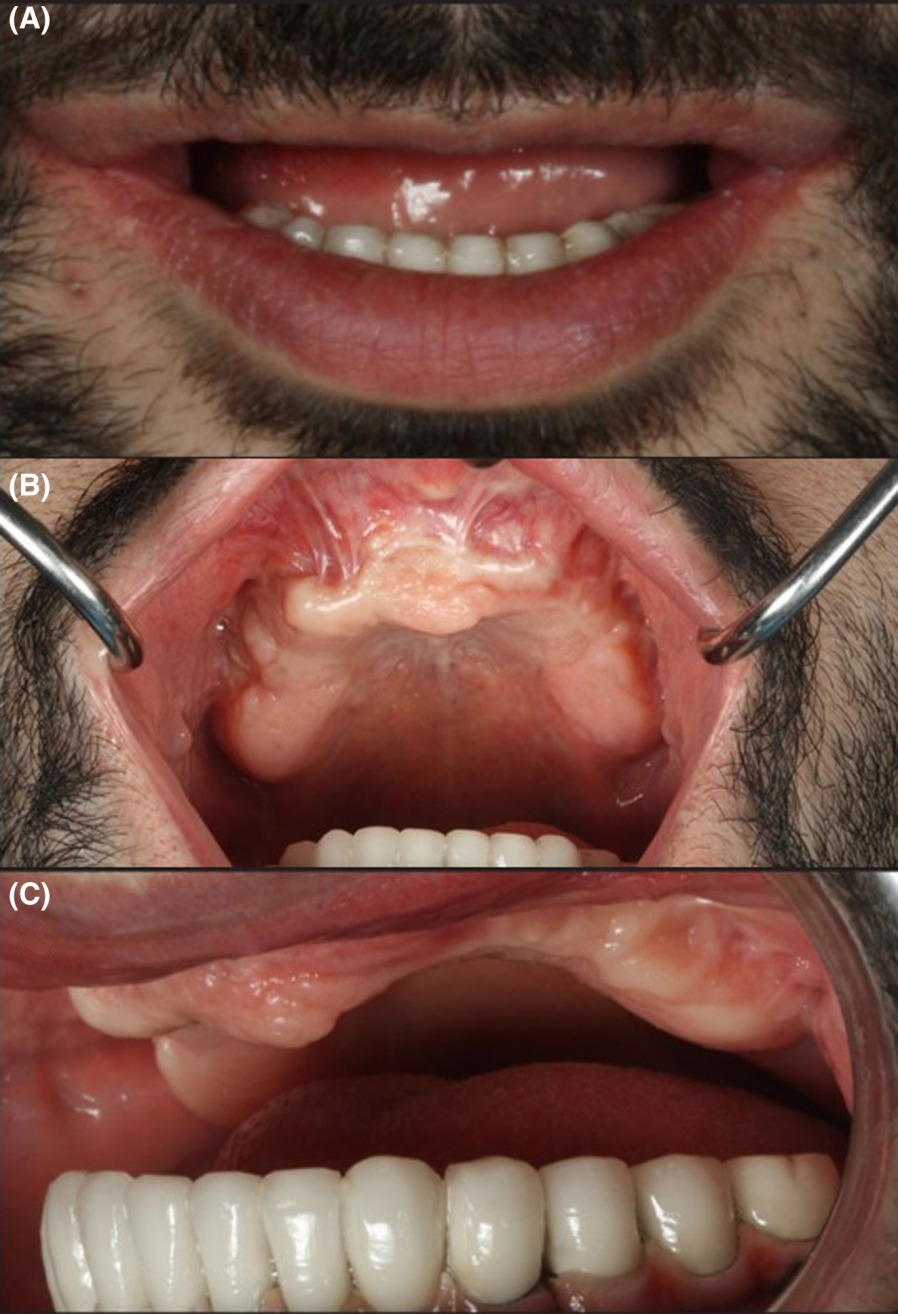
Different views of patient 1 year after the initial implant's failure. A: frontal view, B: occlusal view, C: lateral view.

In 2018, a maxillary edentulous 25‐year‐old male patient was referred to the prosthodontic department of Tehran University of Medical Sciences. His chief complaint was extreme dissatisfaction with appearance, mastication, and speech. A detailed medical, dental, and social history was obtained. The medical history and general physical condition were unremarkable. Dental history showed that the patient lost his teeth 6 years ago because of aggressive periodontitis and received dental rehabilitation by implant‐supported full‐mouth prosthesis, without any kind of bone grafting to increase the alveolar bone quality and quantity. After 5 years, all maxillary implants failed (Figure 1A–C). Therefore, he requested full‐mouth dental replacement.

**FIGURE 2:**
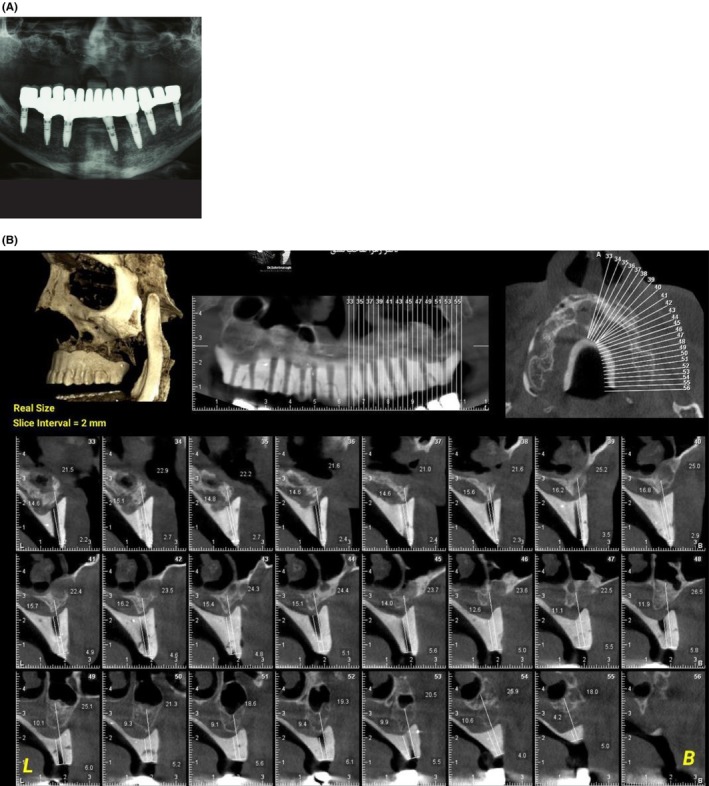
A: Panoramic radiography was taken 1 year after the initial implants failure. B: CBCT of maxilla with radiographic stent.

The clinical and radiographic examination revealed that the peri‐implantitis that led to previous implant failure had caused severe alveolar resorption, and the patient lost a large amount of bone volume (Figure 2A). Thus, placing endosseous implants again without bone grafting was not possible.

Bone graft surgery requires a prolonged healing period added to the waiting period in the implant treatments. The patient was young, and this delay might affect his social and psychological health. Furthermore, restoring the required amount of bone in the patient necessitated an extraoral bone graft, to which the patient did not consent. The patient expressed no inclination toward undergoing iliac bone grafting or extracting bone from the iliac and rib regions. Therefore, we decided to perform SPI reconstruction in the maxilla (Figure 2). After providing a comprehensive explanation of the procedure and its associated risks, we secured informed consent from the patient for the administration of an SPI (subperiosteal implant). The patient also granted consent for the publication of their data.

**FIGURE 3:**
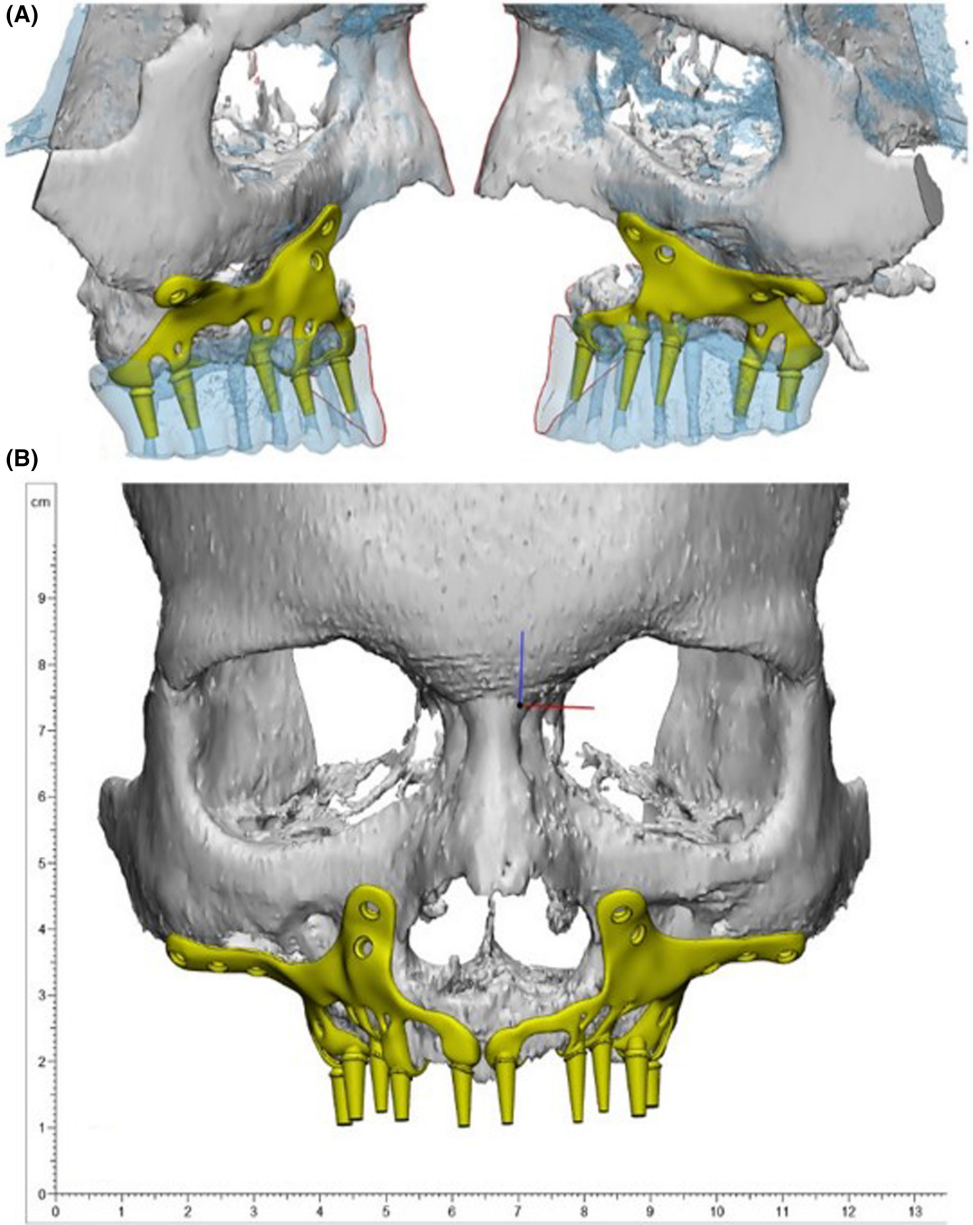
Different views of subperiosteal implants' 3D model design.

To achieve the best function and esthetic possible, first we obtained the primary impressions from both jaws using a prefabricated tray and irreversible hydrocolloid material (Alginate, Zhermack). A special tray was fabricated by auto‐polymerizing acrylic resin and border‐molded using a green modeling plastic impression compound (Kerr Corp.) to achieve a more accurate final impression. The final impression was obtained using zinc oxide eugenol paste (Luralite). A record base for the maxillary arch and a wax rim were created. The alignment of the jaws was recorded based on considerations of esthetics and phonetics. then the maxillary cast was mounted using an arbitrary face bow (Dentatus, Dentatus Ltd.) on a semi‐adjustable articulator (Dentatus A.R.H., Dentatus Ltd.). The mandibular cast was connected to the maxilla using a centric relation record. Anterior tooth set‐up (Vivotac/Orthotak, Ivoclar Vivadent) was accomplished on the mounted casts, and a diagnostic tooth set‐up was performed and tried in. The acrylic teeth and base were poured with barium sulfate (Foshan Xinmei Chemical) containing acrylic resin. This made the teeth opaque so that the occlusal plane and longitudinal axis of teeth could be detected in the CBCT. To establish lucency in the CBCT scan and ascertain the longitudinal axis of the final implant abutments, perforations were made using a fissure tungsten laboratory bur. These perforations were strategically placed at the center of posterior teeth and the cingulum of anterior teeth, reaching from the crest of the maxillary ridge (Figure 3A,B).

**FIGURE 4:**
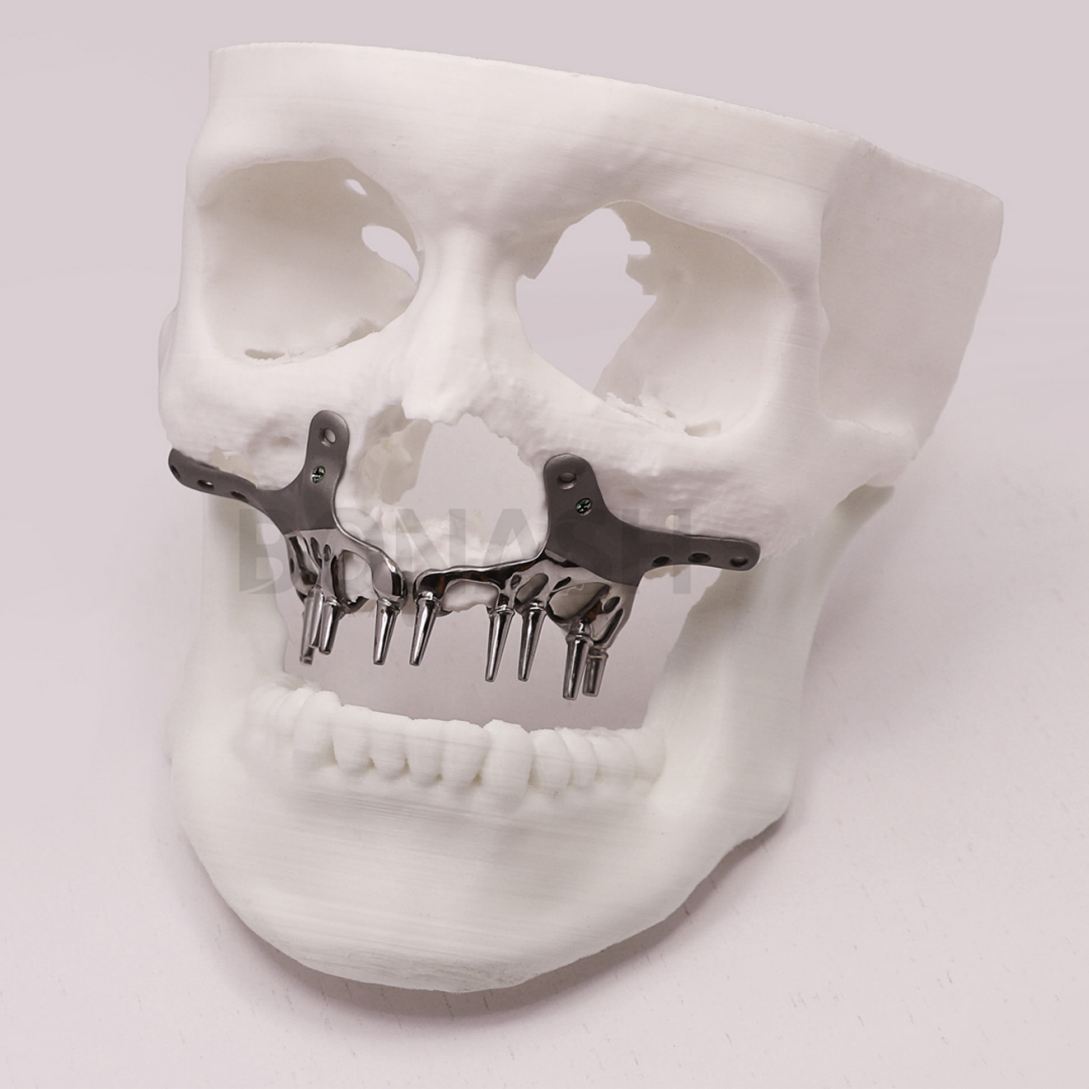
The fabricated SPI model was screwed to a printed skull to evaluate it.

**FIGURE 5:**
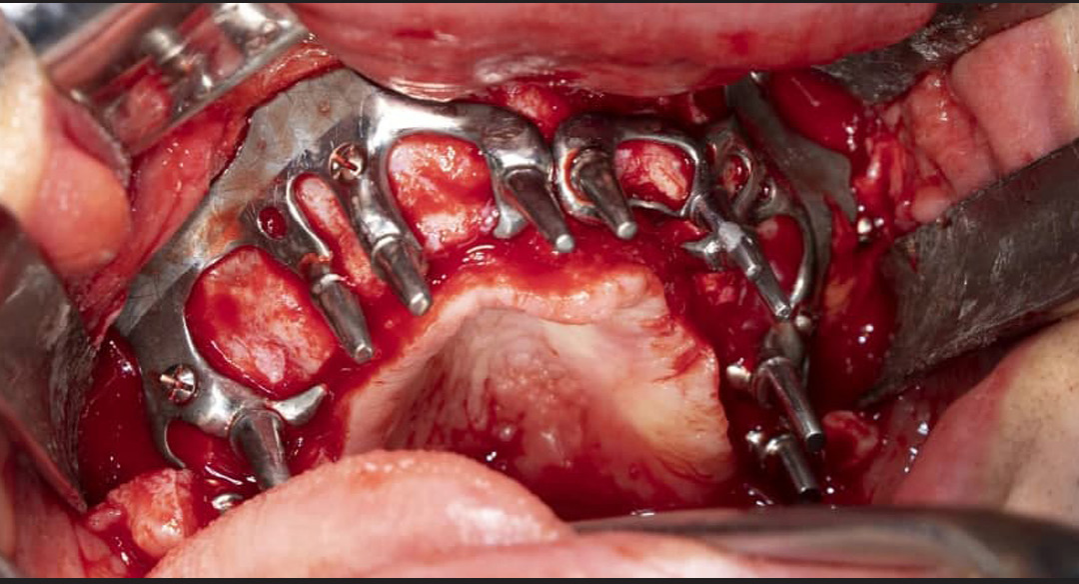
After final confirmation of the implant, an oral surgery was performed to place the implant.

In collaboration with the prosthodontist and surgeon, the engineer undertook the design of the SPI. Consequently, a 3D model was generated using the patient's CT scan data to evaluate the most suitable design and positioning for the SPI. Particularly critical was the meticulous design of the abutments, considering their precise placement and orientation, given the irreversible nature of adjusting abutments after SPI placement in the oral cavity. Consequently, the reverse‐engineering process demanded a high degree of precision, necessitating the accurate determination of abutment location and angle. This determination hinged on the final alignment of the teeth, which was established with regard to both esthetic appeal and functional performance. After carefully planning the implants' placement, the SPI was crafted using the additive manufacturing method, utilizing titanium alloy grade 23 (Ti6AL4V‐ELI, Bonash Company) (Figure 4). Upon completion of the SPI fabrication, it was affixed onto a printed skull model for evaluation. The prosthodontist assessed factors including interocclusal clearance as well as the angles and positions of the abutments (Figure 4). Then it was scanned, and STL data were sent to the laboratory for temporary and final crown fabrication. Subsequently, the SPI was transported to the cleaning area and subjected to sterilization through autoclaving. The patient underwent a surgery performed under general anesthesia to implant the SPI (Figure 5A,B).

**FIGURE 6:**
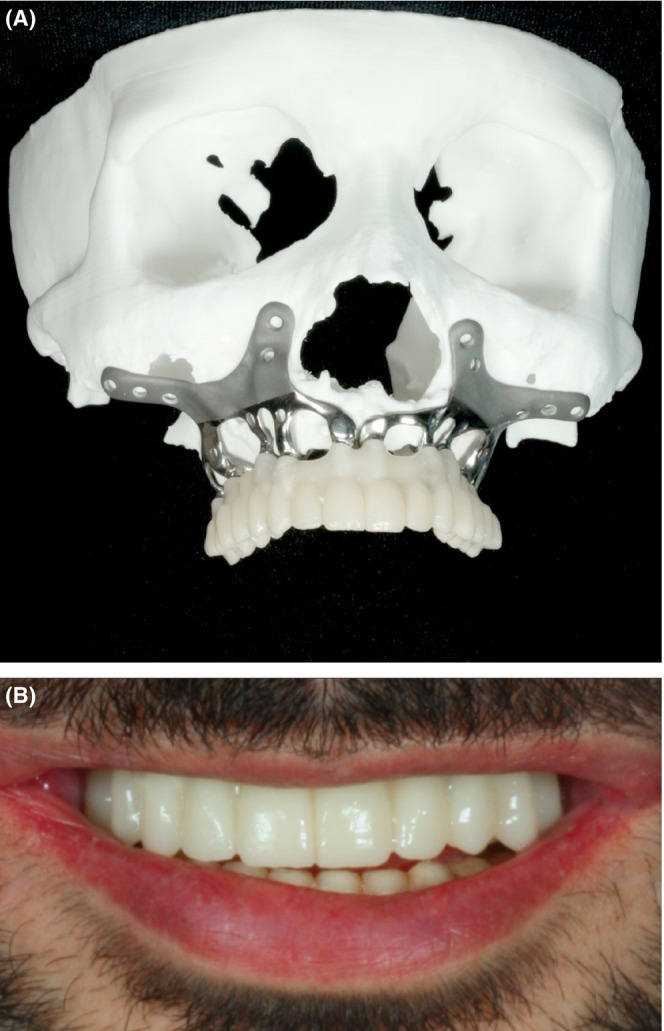
A: Temporary prosthetic design based on 3D printed skull model. B: The temporary prosthetic is cemented in place.

**FIGURE 7:**
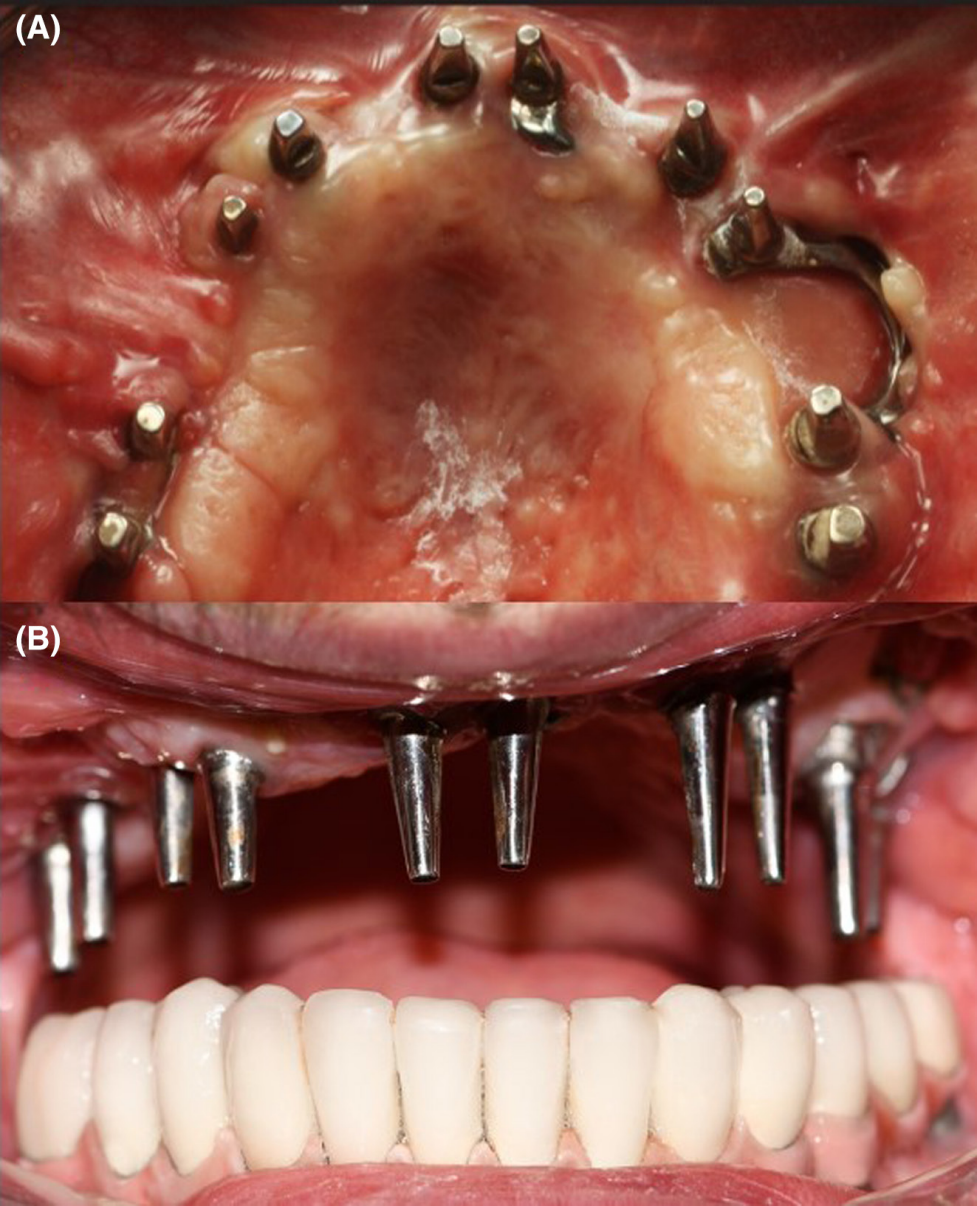
A minor dehiscence occurred in the left premolar area but did not extend over time.

**FIGURE 8:**
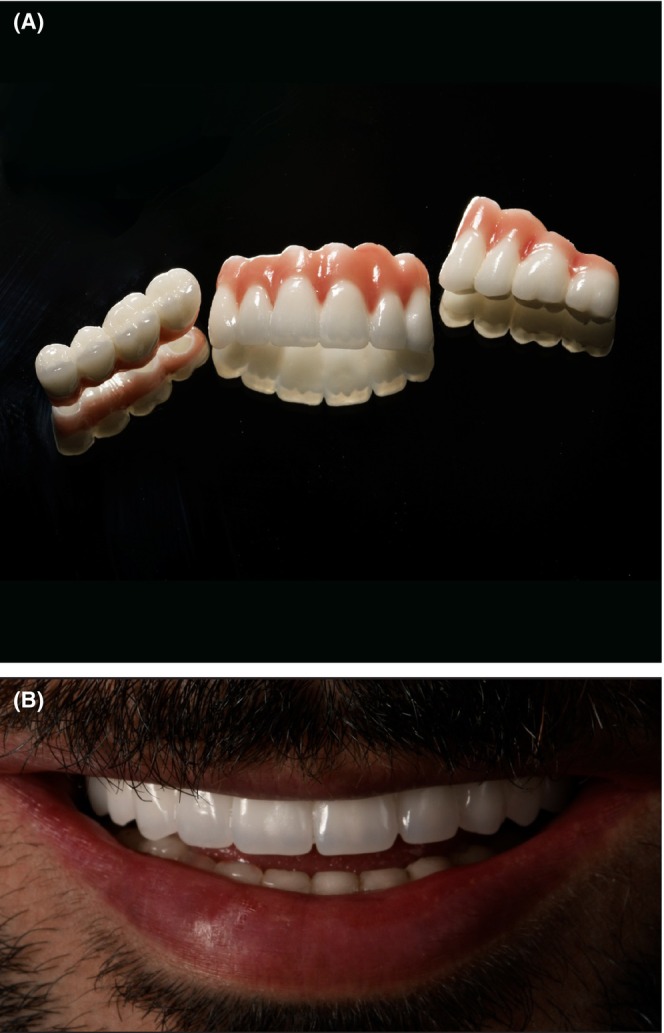
A: The prosthetic design. B: Final prosthetic delivery.

After 2 weeks, a temporary implant‐supported bridge was delivered to the patient (Figure 6A). They were cemented with temporary cement (Temp bond, Kerr) (Figure 6B). A waiting period of 2 weeks was observed before the temporary placement, providing sufficient time for the healing of soft tissues, removal of sutures, and reduction of swelling. This measure was taken as a preventive step to prevent the temporary cement from entering the healing wound. Minor dehiscence was found in the buccal area of the left premolar region and palatal area of the left incisor (Figure 7A,B). However, this dehiscence did not extend in the following sessions. Three months later, a set of three‐part full ceramic computer‐Aided Design / Computer‐Aided Manufacturing (CAD/CAM) bridges was created for the maxillary region, utilizing BL4 shade. These bridges were crafted using digital data acquired from the pre‐surgery SPI scan (Figure 8A). The crowns were inserted into the oral cavity, and the frames' fit was verified. Necessary adjustments were made to the teeth to attain a group function occlusion (TEMP Bond, Kerr; Figure 8B). The implant‐supported crowns were affixed using temporary cement.

**FIGURE 9:**
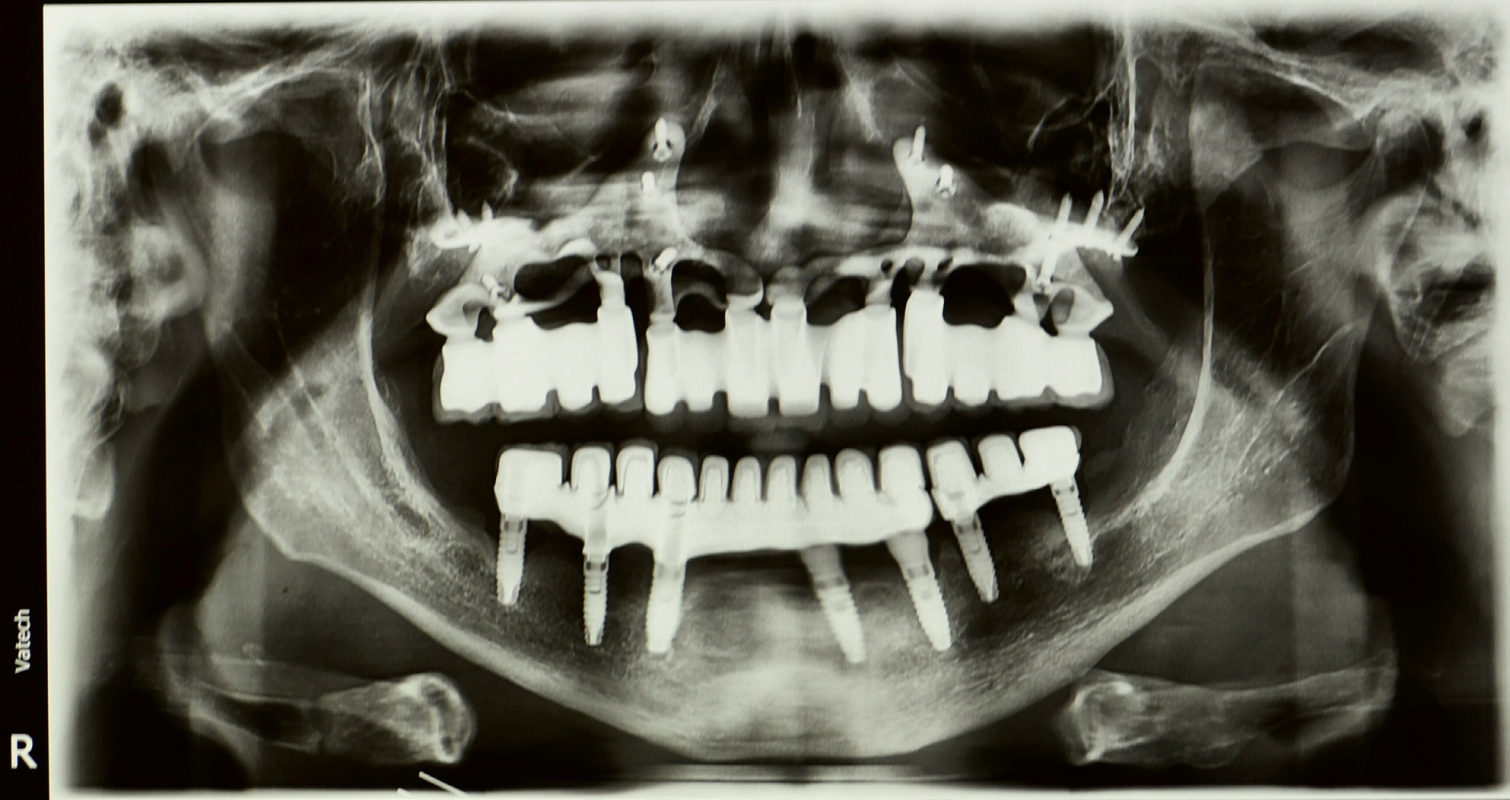
Panoramic radiography showing implant status after 3 years.

**FIGURE 10:**
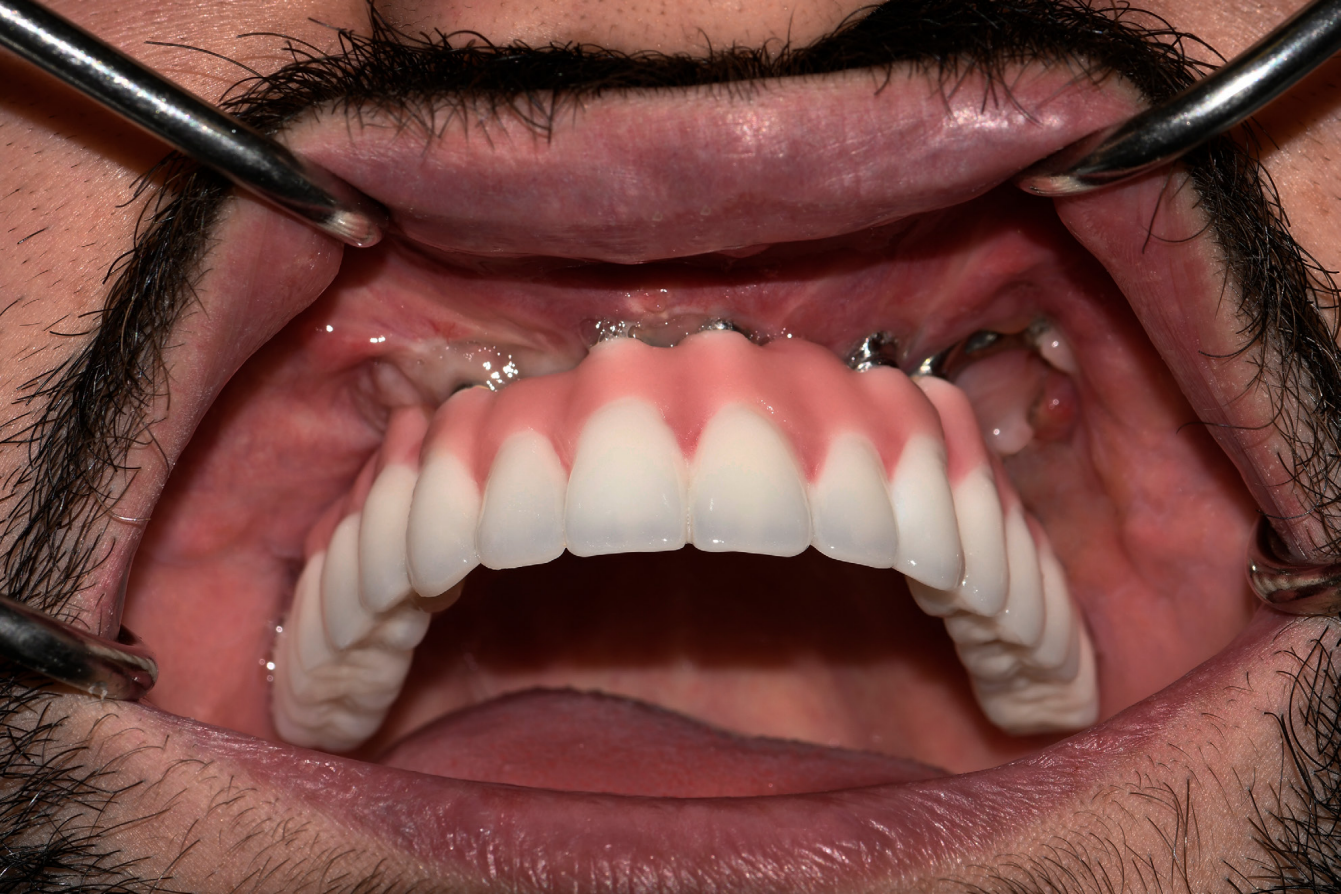
Intraoral view of the prosthesis after 3 years.

We scheduled follow‐up sessions postdelivery, and the patient was instructed to a careful oral hygiene routine consisting of waterjet, teeth brushing, and super floss. Our follow‐up sessions over 3 years showed no progress in the dehiscence in the previous areas or other regions, no signs of fracture, and excellent oral hygiene.Furthermore, the treatment resulted in the patient experiencing satisfaction across aspects of functionality, esthetics, and overall well‐being (Figures 9 and 10).

## 3 DISCUSSION

Since the 1980s, dental endosseous implants have become one of the most common replacements for teeth.^1^ However, the need for adequate bone quality and quantity is not always met. Restoring a resorbed alveolar ridge through dental rehabilitation, irrespective of its origin, necessitates a complex and challenging treatment path. Among the prevailing strategies for addressing this issue, bone grafts stand out as the primary approach for bone replacement. However, bone grafts need a long healing period and have unpredictable results.^4^, ^7^ Furthermore, they can potentially lead to substantial complications. As an illustration, a study indicates that neural complications associated with an intraoral bone graft approach could reach up to 50%.^8^

Recent progress in dental prosthesis technology has enabled a reevaluation of traditional treatments with a diminished risk of complications. Subperiosteal implants (SPIs) have emerged as a viable alternative to various forms of bone grafting and other approaches employed on compromised ridges. With the latest advancements in prosthetic manufacturing, many of the challenges encountered in the past have been effectively surmounted.^2^, ^6^ The results of a retrospective study on 70 patients with SPIs show that after 2 years of follow‐up, the survival rate of SPIs was approximately 96%. In the study, the authors report that only three of the SPIs failed due to untreatable recurrent infections.^9^

In a more recent case series study, the survival rate of 10 SPIs in partially edentulous patients was 100% after 12 months of follow‐up. However, one SPI had a minor immediate postoperative complication managed successfully by antibiotics and pain relievers. The authors believe that the accurate fit of the SPIs was the main reason for the low incidence of complications resulting in a high survival rate.^10^

This report outlines the processes and approaches utilized to create and provide an SPI (subperiosteal implant) for our patient. The patient had lost his teeth at quite a young age due to aggressive periodontitis and received dental endosseous implants as a replacement. Unfortunately, after 5 years, the prosthesis and implants were explanted, leaving him in need of another treatment course. Due to severe alveolar bone resorption, the patient needed an extraoral bone graft to achieve the required amount of bone support for endosseous implants. In addition to the prolonged healing period, he was unwilling to undergo a second surgery. Therefore, we decided to use an SPI for full‐mouth rehabilitation. Our 3‐year follow‐up shows that the SPI is in excellent condition, and the patient is satisfied with the results. While certain areas of implant placement are situated within the alveolar mucosa rather than firmly attached gingiva, we chose to proceed with ongoing monitoring instead of opting for a corrective surgical procedure. In order to mitigate the possible risk of implant rejection, the surgeon chose to avoid additional interventions. As a result, our emphasis shifted toward upholding proper hygiene practices for the patient and adhering to routine follow‐up sessions. Fortunately, there was no observed progression in the dehiscence area.

The SPIs have reemerged, especially during the last decade, and reports of successful SPI dental rehabilitations are increasing thanks to the new manufacturing techniques.^2^ Nevertheless, underestimating the importance of different procedure steps may cause many complications and may eventually lead to implant failure. For instance, using a radiographic stent based on a provisional dental setting helps determine the perfect design and angulation for the attachments. To conclude, SPIs provide an alternative implant solution for cases involving severe bone atrophy. Employing a personalized, prosthesis‐centered reverse‐engineering method, this approach circumvents the need for bone grafting and facilitates immediate functional restoration through a single surgical procedure.

## AUTHOR CONTRIBUTIONS

**Mahnaz Arshad:** Conceptualization; data curation; funding acquisition; investigation; methodology; project administration; resources; supervision; validation; writing – review and editing. **Nourin Khoramshahi:** Data curation; software; validation; visualization; writing – original draft. **Gholamreza Shirani:** Conceptualization; methodology; resources; validation; visualization; writing – review and editing.

## FUNDING INFORMATION

None.

## CONFLICT OF INTEREST STATEMENT

All the authors of this report wish to disclose that there are no financial or other conflicts of interest that might have biased the scientific information in this article.

## CONSENT

The patient has signed an informed written consent form for publishing this report. The consent form was translated to Persian based on the journal's patient consent policies.

## Data Availability

The data that support the findings of this study are available from the corresponding author upon reasonable request.
